# Indian philosophical foundations of spirituality at the end of life

**DOI:** 10.1080/13576275.2017.1351936

**Published:** 2017-07-19

**Authors:** Hamilton Inbadas

**Affiliations:** a School of Interdisciplinary Studies, University of Glasgow, Dumfries, UK

**Keywords:** Spirituality, Indian philosophy, end of life, palliative care, death

## Abstract

Growing understanding of spirituality at the end of life demands more theoretical research on the subject. Empirical studies have highlighted the need for exploring philosophical and cultural concepts to facilitate a fuller understanding of spirituality at the end of life. This paper explores Indian philosophy to inform the conceptualisation of spirituality at the end of life in the Indian context. Three key themes from discourses on spirituality at the end of life have been analysed: the concept of the human person, the purpose of life and the meaning of death. The human person is from and of the Divine, eternal and is capable of cognition and experience. The purpose of human life is to unite with the ultimate Reality, the Divine, by living life righteously according to prescribed ways and by achieving detachment from the illusion of the world. Death is part of life and not that which ends it. The moment of death is an opportunity for the ultimate transformation, Moksha. Analysing these philosophical foundations can provide the contextual frame for understanding the spiritual needs of palliative care patients and their families and the possibility of developing culturally relevant approaches to providing spiritual care at the end of life.

Attempts to explore and understand the concept of spirituality and its implications for end of life care are on the increase around the world. Indian palliative care community with its 30-year history has demonstrated its commitment to further its knowledge base of the spiritual aspect of palliative care through engaging in research studies. The first systematic review of studies related to spirituality at the end of life in India was published in 2015, which analysed 39 empirical studies and 18 reviews, opinion and discussion papers (Gielen, Bhatnagar, & Chaturvedi, 2016). While promoting an understanding of spirituality based on patient and staff perspectives, these studies have highlighted the need for unpacking the historical and cultural meanings of concepts that shaped the understanding of spirituality in the Indian context. Studies have demonstrated that Indian philosophical and religious concepts such as *Karma*, *dharma*, *ātman* and the notion of rebirth, for example, have a considerable influence on Indian perceptions of spirituality at the end of life (Chattopadhyay & Simon, 2008; Desai, 1988). Establishing an overview of the philosophical concepts is crucial for understanding their influence on perceptions of spirituality.

Despite the historical link between spirituality and theology/philosophy, the recent approaches to studying spirituality lack attention to philosophical and theological underpinnings of spirituality. Most studies on spirituality at the end of life seek empirical evidence based on interviews and the historical and cultural components of spirituality are often left unexplored (Inbadas, 2016). Far less theoretical research is found on the subject and consequently, the philosophical basis of spirituality attract little attention (Cobb, Dowrick, & Lloyd-Williams, 2012). Empirical evidence makes significant contribution in identifying ‘what’ constitutes spirituality for a given population. However, without theoretical exploration of concepts and philosophical thoughts, answering the questions of ‘why’ and ‘how’ various values and principles gain significance to constitute the content of spirituality in particular contexts remains challenging to answer.

Philosophy constitutes one of the key conceptual areas necessary for understanding spirituality (Cobb, 2001). There are a few examples of engagement with philosophical concepts drawing from modern western philosophers to understand and explain spirituality and its relationship and implications for health and health care (Eseadi et al., 2015; Goddard, 1995; Henery, 2003; Patterson, 1998; Pesut & Sawatzky, 2006). In the context of end of life care in India, several avenues for potential engagements with the Indian philosophical fabric can be identified. *Vedic* texts of ancient India consider the care of the dying as one of life’s major responsibilities (Davidson, 1988). Movements within Hinduism (e.g. *bhaktimarga*) have specific teachings about how to approach end of life along with teachings on monastic life, celibacy, etc., which are also found in classical literature (Firth, 2005). While the *Vedic* traditions represent the philosophical streams of the north India, resources from the philosophical traditions of *Siddhars* offer the *Dravidian* perspective from the southern part of the country (Ganapathy, 1993).

Empirical studies from the Indian context indicate the influence of Indian philosophical thoughts on current notions of spirituality at the end of life. Indian philosophical and religious concepts often appear in interviews with palliative care patients and palliative care professionals (Elsner, Schmidt, Rajagopal, Radbruch, & Pestinger, 2012; Manghrani & Kapadia, 2006; Simha, Noble, & Chaturvedi, 2013). Considering the profound relationship between Indian spirituality and Indian philosophy, exploring the philosophical foundations of aspects of Indian thought and worldview that impact the understanding of spirituality at the end of life becomes crucial. This paper, therefore, aims to present a review of selected philosophical and cultural literature from the Indian context to establish the philosophical foundations of spirituality specific to the end of life.

It is necessary to highlight some features of Indian philosophy and its relationship to spirituality. India has a rich philosophical heritage over several thousands of years. These ancient philosophical traditions had many schools of thoughts and had substantial body of intellectual argumentation about reality, the human person and their inter-relatedness. Jitendra Nath Mohanty, a renowned Indian philosopher, defines philosophy as reflections on experiences of human persons involved in time and history – about the self, others and the world (Mohanty, 1993). It is important to recognise that there is no ‘Indian philosophy’ that represents the whole of the Indian subcontinent. There are specific regional traditions that represent the historical and philosophical worldview of parts of the country and over several periods of time. For example, *Vedic* philosophy captures the *Aryan* cultural tenets predominant in the northern parts of the country and *Shaiva Siddhānta* is a tradition that has a specific Tamil lineage referring to the life and culture in South India, popularly known as the *Dravidian* culture (Ganapathy, 1993; Kesavan, 1997). Some of these philosophical traditions find their expressions in the forms of classical poetry. In Tamil literature, for example, *Thirukkural* and *Sanga ilakkiyankal* are among the most notable examples for this.

Indian scholars have shown that philosophy, religion and medicine belonged together in ancient India and that they together captured Indian worldview (Ganeri, 2007; Kutumbiah, 1999). Rajendra Prasad’s definition of Indian philosophy illustrates the relationship between spirituality and philosophy in India: Indian philosophy ‘is a reflective, reasoned account given by classical thinkers, of their spiritual intuitions of what the ultimate, most basic reality is’ (Prasad, 2008, p. 27). Another scholar, Dasgupta (1922) in his historical overview of all the major Indian philosophical traditions, observes that despite variations between the different traditions, three main doctrines remain commonly significant for all of them: the doctrines of *karma* – the principle of causality, *mukti* – release from the cycle of life in this world, and soul, *ātma* – the ‘inner-self’ of the human person. These common doctrines of Indian philosophical traditions also testify to the relationship between philosophy and spirituality in the Indian context.

Recognising that this affiliation is fundamental to understanding the philosophical foundations of Indian spirituality at the end of life, I undertook a review of philosophical and classical Indian literature that shed light on relevant themes. Given the complexity and expanse of Indian philosophy spread over thousands of years of oral, visual and written traditions this paper does not claim to offer a comprehensive review of all philosophical traditions. Much of these reviews involved engagements with strands of *Vedantic* and Dravidian philosophical traditions. The key points that emerge may have resonance with others philosophical traditions, both of Indian origin and of wider contexts.

Spirituality as an ‘essence of the human person’ and its close association with the ‘meaning and purpose of life’ feature as the most prominent themes in defining spirituality in end of life care research (Cobb, 2001; Fegg et al., 2010; Hermsen & ten Have, 2004; Kellehear, 2000; Narayanasamy, 2006; Swinton, 2010; Swinton & Narayanasamy, 2002). In addition, the context of end of life can be considerably influenced by philosophical and cultural meanings of death. I therefore engage in an Indian philosophical exploration of the three concepts that seem to be the key in shaping the understanding of spirituality at the end of life, namely: the concept of the human person, the purpose of human life and the understanding of death.

## Concept of the human person

The human person is often referred to as *ātman*, (soul), meaning ‘the self’, the ‘inner spirit’, the person’s true self or the inner person, in Indian philosophical literature. The perception of the human person is one of the most principal concepts in the Indian philosophical traditions. Acknowledging that there have been many and varied philosophical constructions concerning the notion of the human person, I present a selection of them, which several Indian scholars have identified as considerably influential in Indian thought. One of the ways to enter into the discussion about the concept of the human person is by exploring the theories of the origins of the universe according to the Indian philosophical traditions. The description of the origins of the universe as depicted in *Chandogyopanisad*, part of the *Vedic* philosophy, one of the earliest known philosophical traditions in India, is a typical example:

In the beginning there was Existence alone – One only, without a second. He, the One [*Brahman*; Sanskrit word to represent ‘the ultimate reality underlying all phenomena’, often translated as ‘God’], thought to himself: ‘Let me be many, let me grow forth.’ Thus out of himself he projected the universe, and having projected out of himself the universe, he entered into every being. All that is has its self in Him alone. Of all things He is the subtle essence. He is the truth. He is the Self. And that … THAT ART THOU! (Chandogya Upanishad VI cited in Gupta, 2002, p. 45)

This description of *Vedic* cosmology, in common with other Indian philosophical traditions, conveys a number of prepositions regarding the concept of the human person. I identify two of them that I consider are relevant for the understanding of spirituality at the end of life: firstly, that the human person, *ātman*, soul is directly connected with the ultimate reality, God, just as every other being in the universe is, because everything came to existence from God, the ‘eternal Existence’. *Vedic* philosophy thus presents the notion of ‘the reality of cosmic unity’, originating from one and the same Reality, where one is constantly interconnected with everything and everyone else, which are all of the same substance, originating from *Brahman* (Supreme Divinity).

Krishnanda explained the connectedness of the individual human person to the rest of the universe using an illustration from the *Upanishads*, of the ocean and its waves. Individuals are like waves in an ocean forming the crest of the body of the ocean, which is the rest of the cosmic order:

The fact of the organic connection of the individual with the *Vaishvanara* (Universal or Cosmic Self) implies that there are cosmical aspects operating even in the individual, just as everything that is in the ocean is also in the wave, notwithstanding the difference between the crest called the wave and the body which is the ocean. (Krishnananda, 1977, p. 75)

Secondly, the *Vedic* philosophy, as well as most Indian traditions hold that the human spirit, *ātman*, is eternal (Monier-Williams, 1974). Because the soul is from the Eternal One, the human soul also takes the characteristic of being eternal – they exist before they take the form of a human person and after, because the human spirit is part of the Eternal one, *Brahman*:

*Ātman*, the spirit of vision, is never born and never dies … the Eternal in man cannot die. (Gupta, 2002, p. 50)

The following quote from *Bhavad Gita*, further illustrate the belief that the human soul is part of the divine and because it is part of the Divine, it is also eternal:

An eternal portion of Myself [God] having become a living soul in the world of life, draws to itself the five senses with the mind for the sixth, abiding in Nature – (The Bhagavad Gita, Chapter 15, Verse 7 in Śankarāchārya, 1983, p. 497)

These prepositions clearly indicate that the predominant discussion about the human person in Indian philosophy is about the *ātman*, the soul of the human person. Another significant notion in Indian thought regarding the human person is that *Ātman*, the soul, is central to the understanding of epistemology: Indian philosophers hold that the soul, *ātman,* as ‘the spirit of vision’ represents the capacity of the ‘self’ of cognition or awareness, to ‘see and to know’ (Chakrabarti, 1999). The possibility of ‘knowing’ is given to the human person thorough the self, the soul.

The deliberations of the Indian philosophical traditions on the relationship between the body and the soul further demonstrate the significance of the soul in the understanding of the human person in Indian philosophical thinking. Many traditions have laid much emphasis on the relationship between the soul and the body as the ‘embodied self’. Buddhist philosophers give the analogy of a chariot to describe this relationship. They explain that just as neither the different parts of a chariot, nor combination of them make a chariot, the ‘self’ cannot be perceived independent of the body and the cosmos in which it lives (Conze, 1959). In this view, the body and the soul are seen as part of each other making the whole – the person. The view of most Indian philosophical traditions on the human person is that of an ‘embodied self’, where the body is understood as the part of the person that makes ‘experiencing’ possible and the ‘self’ making sense of that experience. Philosophers from one of the oldest Indian traditions, the *Sā Khya* School, explain this using the analogy of the chariot and the charioteer:

Just as a chariot requires a charioteer, co-ordination of our experiences reveals a consciousness which makes that co-ordination possible (Mahalingam, 2002, p. 146)

Indian philosophers have dedicated much discussion on the role of the body and the embodied nature of the human person in achieving *mukti,* the eternal liberation. *Upanishad*, an important Hindu scripture emphasises the thought that this eternal liberation is the union of the soul with its true nature, the Divine and that this union is possible only after the soul has departed from this earthly life, form the body:

The Soul of mine within the heart, this is *Brahma*. Into him I shall enter on departing hence. – Chandogya Upanishad, 3.14: 2–4 (Cited in Adiswarananda, 2007, p. 206–207)

The concept of ‘the embodied nature of the human person’ follows that the body, through the experiences of ‘ignorance’ (of the soul’s true, divine essence and nature), pain and suffering, persist as a hindrance for the soul to attain *mukti* or *nirvana*, the ultimate liberation from the bondage of the body to the possibility of the union with the soul’s true nature, the Eternal one, God (Hiriyanna, 1995; King, 1999).

Contrary to the notion that the embodied status of the human person is a hindrance to attaining union with the divine, *Dravidian* philosophical traditions from south India reveal a positive view on the body. For example, the *Siddar* philosophy considered the human body as ‘the dwelling place’ of the Divine in the form of the spirit, the soul of the person. According to this school of thought the body is not an obstacle, but a facilitator. The body is considered the vehicle of spiritual evolution towards achieving *moksha*, the realisation of the Divine within (Ganapathy, 1993). Philosophers from this tradition also believed that this realisation can be achieved while still being in this embodied self and not after the death of the body. Ascertaining the positive view of the body, *Avvaiyar*, a Tamil poet and philosopher who lived in the *sangam* period (1 – second-century CE), articulated that the only use of this embodiment for the soul is to realise the Divine within the body (Ārumuganāvalar, 1987). In another poem she suggested that by achieving immaculate principles of life in one’s mind, the inner self, his/her body will manifest the Divine, thus offering a view of the body that facilitates the transcendence to expressing divine virtues (Ārumuganāvalar, 1987).

It is, therefore, evident that the notion of the human person is expressed as the ‘self’, the indwelling spirit, which is from and of the Divine. It is this ‘self’, the soul, that is capable of cognition that makes experience possible through being embodied in this world. Despite the differences in their approach to the body, the various schools of thought maintain that the soul is at the centre of all the deliberations about the human person. They also hold that because the ‘self’ is immortal and a portion of the divine, the ultimate liberation for the soul is to be united with the Divine. This ultimate liberation is considered the purpose of human life.

## The purpose of human life

Perceptions of the purpose of human life in the Indian philosophical traditions have substantial links to the conception of the human person. All major schools of Indian thought suggest that the purpose of human life is to achieve the union of the ‘self’, the soul with the Divine. The *Vedic* tradition holds that the human life is the opportunity for spiritual improvement in order to achieve the ultimate goal of realising the fundamental truth about oneself, which is the realisation that I am the Eternal One, ‘Aham Brahmāsmi’ (meaning: ‘the soul and the world are one’, Dalal, 2009); that is, to achieve a state of self-realisation, where the ‘self’, the soul is indistinguishably identical and one with the *Brhaman,* the Divine. This realisation is the ultimate liberation of the human soul, which is also commonly known as *mukti* or *moksha*.

Different schools have laid emphasis on different aspects of human life that lead to achieving this liberation, *moksha*. According to the orthodox Indian philosophical view it is in achieving perfection through three goals of life: *artha* (prosperity), *kāma* (desire) and *dharma* (righteous living) that the fourth and the ultimate goal of life, *moksha* can be attained (Mohanty, 2001). However, most schools consider *dharma* (righteous living) as the foremost of the three goals. They regard *dharma* as the canon that gives moral foundation for human life: to be honest, attempt to fulfil moral obligations, to be genuinely concerned about others, etc. (Vade, 2002). *Dharma*, in addition to facilitating the attainment of the ultimate liberation of the individual soul, also maintains social stability and harmony. For example, *dharma* implies responsibility of the ‘self’ in the society is the ‘moral obligation’ a father has to his children; a son has to his parents, and everyone in the society to everyone else. The self being able to accomplish these moral obligations is important for the attainment of the ultimate liberation of the soul. Scholars have argued that the role of rituals in the Indian society was to operationalise *dharma* – providing a framework for executing these moral obligations. They also indicate that these rituals were propagated through oral poetry in communities and helped consolidation of social values (Subramaniyan, 1982).

It is evident that many of the Indian philosophical traditions emphasise ‘living a disciplined life with righteous actions’ is the way to achieve the ultimate purpose of human life, the ultimate liberation, *moksha*. Demonstrating a similar understanding, Sai Baba, (Baba, 1976) another renowned Hindu spiritual leader, holds that ultimate goal of life is to attain the union with the divine through a ‘good and peaceful death’:

We must recognise the truth that all the *Sādhanā* (Sādhanā refers to a disciplined life undertaken towards a goal. (Iyengar, 2004) that we do is not for the sake of a pleasurable life but for a good and peaceful death. All the great saints and *yogis* [sage] direct all their prayers towards aspiring for an easy and good death, and they pray that they be enabled to merge into the Lord easily. Our attempts should be directed to the objective of ultimately merging with divinity. This is the sacred lesson that is contained in the *Shanthiparva* (‘The Book of Peace’, is one of eighteen books of the Indian Epic *Mahabharata.* Baba, 1976, p. 132)

Philosophers who follow this thought argue that the righteous actions of disciplined living of human persons bring a good and pleasurable life both for oneself and for others. However, the purpose of living a disciplined life is not to be limited to the good and pleasurable life for all here in this life. It is so that the self will attain the ultimate purpose of human life, *moksha*, the union with the Divine.

The understanding of the concept of *karma* is important in this regard. *Karma* is a significant and complex concept in the Indian philosophy that governs the perception of ‘duty’ (Konwar, 2013). Ghosh (2014), among many others, indicate that *Karma* – action or work (resultant of previous actions and having implications for the future of the soul) – and *dharma* – accomplishing duties in the ‘right way’ – embody the way Indians make sense of the present as well as the possibility of the achievement of the union with the Divine. Many Indian philosophical traditions commonly hold the belief that being able to complete ones duties destined to a person in this life (*Karma*) in the right way (*dharma*) helps the ‘self’ achieve the ultimate goal of liberating their soul from the cycle of rebirth to be united with the divine (Mahadevan, 1953). The purpose of human life, therefore, is to complete the responsibilities, which are moral obligations for the soul, in righteous ways.

Those who conduct their lives through meditation in this manner are liberated from the bondage of *karma*. They pass through the stages of ascent leading to the higher regions of life, ultimately landing in *Brahma*-*loka*, or the realm of the Creator, for the purpose of ultimate liberation, or salvation; otherwise, there is return, once again, by way of reincarnation, or rebirth (Krishnananda, 1977, p. 47)

The concept of ‘rebirth’ is a significant and popular notion in Indian thought, which is connected with *karma*. After the death of a living being, depending on the *karma* of the ‘self’, *jiva*, the immortal essence or soul of the living being, will take the form of another life. Scholars clarify that the soul takes the form of life in this world several times before it can achieve the ultimate liberation of the union with the Divine:

The self may have gone through many lives before the present one. For an eternal self, changing bodies in different lifetimes and moving from an old body to a new body is like a person discarding worn out clothes and wearing a new one. (Chakrabarti, 1999, p. 156)

The purpose of human life is to use the opportunity of living as a human person to live a righteous life, fulfilling all moral obligations in the right way and thus to try not to get back into another life or rebirth in this world, but to liberate oneself from the cycle of rebirth and to be united with the Divine.

While the thoughts expressed so far signified an action orientated approach to achieving the purpose of life, in terms of ‘doing the right thing’, others focus on having a compassionate heart and ‘God- thought’. *Thiruvalluvar*, a first-century Tamil poet and philosopher articulated in one of his couplets that living a life with a compassionate heart will liberate one from the cycle of rebirth (*Thirukkural*, p. 243). Translated, this couplet means: those with graceful and compassionate hearts will not [re]enter this world of darkness and evil. Similarly, in his commentary on Bhagavad Gita, Prabhupada (1983) a renowned scholar of *Vaishnavite* school of thought, emphasises the importance of God-thought not just at the very end of life but throughout one’s life:

The word *smaran* (‘remembering’) is important. Remembrance of *Krishna* (one of the most popular gods in the Hindu religion, Krishna is the eighth incarnation of Lord Vishnu) is not possible for the impure soul who has not practiced Krishna consciousness in devotional service. Therefore one should practice *Krishna* consciousness from the very beginning of life. If one wants to achieve success at the end of his life, the process of remembering Krishna is essential. Therefore one should constantly, incessantly chant the *maha*-*mantra* – *Hare Krishna, Hare Krishna*, *Krishna Krishna, Hare Hare/ Hare Rama, Hare Rama, Rama Rama, Hare Hare*. (Prabhupada, 1983, pp. 370–71)

Swami Prabhupada implies that only through chanting the name of God the soul can be made pure and with the right thoughts. According to his thoughts, ‘God consciousness’, or God-thought has to be part of a person’s entire life and that this God-thought is associated with purity of the soul, which helps people to achieve ‘success’ at the end of life, which is the union with the Divine.

Another important aspect of understanding the purpose of human life is presented in the notion of the temporality of human life. The most popular concept in this regard relates to the notion of *maya*, often translated as ‘illusion’. Vivekananda, one of the important philosophers of the Neo-*Vedanta* school of Indian thought refers to *maya* as the human person’s clinging to life, one’s inability to release oneself from the bondage of life in this world (Vivekananda, 1991). He explained that the real freedom is liberating oneself from this clinging and bondage to life to the realisation of the union with the Divine, which according to the *Vedanta* school is within the human being, the soul. This freedom is about detachment from the illusion of the life in this world in order to be united with the Reality, the Divine.

It is in this context that the ideas of ‘detachment’ and ‘renouncing earthly pleasures’ become crucial. Classical Indian texts have numerous references to sages (*Vāndaprasātha*) who lived as hermits, after giving up all desires of life. These sages withdrew from ‘the illusion of the world’ after completing all duties required of the person (Nilakanta Sāstri, 1972). A classical Tamil poem describes the human life as a ‘canoe made of straw’ trying to cross ‘the ocean of fire’, implying that the human body and the physical world we live in are temporal and will be destroyed. The poem appeals that one should remember that death is awaiting at the corner and should not depend on the body and the physical world. The poet implies that detachment from the body and physical world is the way to achieve what it takes to embrace death to this world, and the realisation of the ultimate purpose of human life (*Nīthi nūl* 423). This poem is a typical example of the thoughts expressed in many other poems and writings found in Tamil literature right from the classical period. They all reflect this Indian philosophy of human life – detachment from the physical world towards being at peace at death. In the words of Swami Sivananda, a Hindu spiritual teacher: ‘You must get yourself buried in God. Then only you shall live. You gain by losing. You live by dying’ (Singh, 1983, p. 44). He emphasised the view that losing or detaching from everything from this world and life is the way to attain God, which is gain for the human soul.

The purpose of human life, according to Indian thought, is to unite with the ultimate Reality, the Divine. I have identified several ways prescribed by different schools of thought to achieve this purpose. They include: living a virtuous life, completing the moral obligations in the ‘right way’, having a compassionate heart, having God-thought and by renouncing and detaching from the world of illusion. These ways offer the possibility of achieving the ultimate purpose of human life, which is to realise the potential and to unite with the Divine, the ultimate Reality.

## The meaning of death

The understanding of the meaning of death in Indian thought is characterised by the concept of the human person and the understanding of the purpose of life, which I have discussed in the previous sections. In the Indian philosophical understanding, ‘death is not the opposite of life – it is the opposite of birth. The two events simply mark a passage’ (Desai, 1988, p. 251). Death is that event where the breath of life leaves behind a worn out body, just like we get rid of worn out clothes (Adikalaar, 1975). Similarly, *Thiruvalluvar* a Tamil poet and philosopher from the early part of the first millennium wrote in *Thirukkural* 339 (Reddiyar, 2001, p. 66): ‘death is like a deep slumber and birth like waking from that sleep’ (translated). The poet reiterates the immortality of the soul and its existence before birth and after death. At birth, the soul wakes up to a new life as an embodied self and at death it goes to sleep leaving the body behind. The following statements from *Krishnananda*, a renowned Indian philosopher, capture the classical Indian understanding of death:

When the span of life is finished, there is what we call the death of the body, the extrication of the *prna* (breath of life) from the individual embodiment. (Krishnananda, 1977, p. 47)

What we call death is the departing of life from a particular body. So death is not the death of the life principle itself. *Na jivo mriyata* – life itself does not die. The vitality is transferred from one location to another. It is withdrawn from a particular formation. (Krishnananda, 1977, p. 147)

From these and many other analogies and metaphors that are used to explain the philosophical understanding of death, it can be recognised that death in the Indian thought is much more about the soul than about the body becoming dead. It is more about the breath of life – the soul – continuing its journey, leaving the body behind in order to unite with the divine. Death of a human being, according to most schools of Indian thought, can be the final passage for the soul, which may have had many births and rebirths, if the soul has achieved the ultimate purpose of human life. Because death of a human thus potentially denotes the ultimate liberation of the soul, *Vivekananda*, a nineteenth-century philosopher of the *Vedanta* school, remarks that the death of a human being brings the experience of joy to the soul:

[The] final transcendence of the process of birth, and hence a freedom from body-bondage … does not merely provide a negative happiness of freedom from miseries, it is a state of positive joy (Lal, 1978, 29–30)

Since this liberation from the bondage of the embodied self and the union with the Divine mark the ultimate purpose of human life, *Vivekananda* clarifies that death, which marks this liberation from the attachment to the body and attainment of the union with God, is the opportunity where the soul merges with and becomes one with its true self, the ultimate Reality.

The understanding of death as potential transition facilitating union with the Divine is a significant aspect of the meaning of death in Indian thought. The essence of this understanding can be found in *Bhagavad Gita*, one of the most important Hindu scriptures (Bhagavad Gita 8, p. 5):

And whoever, at the end of his life, quits his body remembering Me (God) alone at once attains My nature. Of this there is no doubt. (Prabhupada, 1983, p. 372)

This verse clearly emphasises the importance of ‘remembering God alone’ at the time of death. One should have dealt with or detached oneself from everything else in life so that s/he has only one thought – the thought of God at the time of death. Such renunciation of human life as well as the world and the unified God-thought, according to most schools of Indian thought is the only way to realise one’s true nature, the realisation that ‘I am part of the Divine’. A well-known Hindu scripture *Bhagvat Puran* cites a story from ancient times where a sage, *Shukmuni*, advises the king how to prepare for his death. In his advice the sage emphasised the difference between the mortal body and the immortality of the ‘Self,’ the human soul and draws the king’s attention ‘to know’, i.e. to realise the true nature of his soul:

When you know that your true nature, ‘Self’, is divine and immortal or you are assured that God is always with you, there is nothing to fear. (Cited in Mehta, 2013, p. 512)

Fundamental to all these thoughts on the subject of death in the Indian understanding is that death is not the end of life, but rather a moment of transition where the ‘self’, the soul of the person leaves the body behind. When doing so, if the self also renounces all the illusions and attachments of this earthly life, and is able to have only God-thought at the time of death, then the soul will attain the realisation of its true self, the union with the Divine. To use an analogy from Shankara, the most influential philosopher of the *advaita* school, death is when the ‘divine spark’ within the human person can merge with the ‘Divine flame’ (Chattopadhyaya, 2000).

Most of the Indian literature that deal with the understanding of death refer to the state of the soul and its transition to being united with the divine. However, in my review I also noticed that some literature dealt with ‘preparing for death’ with much importance. The following verse from *The Bhagavad Gita* specifies why being prepared for death was given such high importance:

Remembering whatever being one gives up the body at the end, that very being one reaches, O Arjuna! Ever conforming to that being. (The Bhagavad Gita, Chapter 8, Verse 6 in Śankarāchārya, 1983, p. 277)

In the earlier sections I captured the notion that the purpose of human life is to attain the union with the Divine, or the merger with the Eternal one. This verse from *Gita* demands that what remains in the mind of the person at the time of death affects the prospects of this ultimate liberation of the soul. I have also indicated that having God-thought at the time of death was considered crucial to have this deliverance. Therefore, Gita implies that the mind of the person should be free from all worries and desires of this world in preparation – i.e. to have such a state of mind at the time of death, through which the soul’s unequivocal thoughts and awareness is on God.

I found that the experience of fulfilment at the time of death is connected with the idea of preparation for death. Several important Indian texts illustrated the notion of the experience of fulfilment and peace at the time of death. India’s first Nobel laureate Rabindranath Tagore (1861–1941), a poet, philosopher, artist, playwright, composer and novelist expressed his views on death as a fulfilment of life in the following poem (*Gitanjali* 91):

O thou the last fulfilment of life,
Death, my death, come and whisper to me!
Day after day I have kept watch for thee;
for thee have I borne the joys and pangs of life.
All that I am, that I have, that I hope
and all my love have ever flowed towards thee
in depth of secrecy.
One final glance from thine eyes
and my life will be ever thine own.
The flowers have been woven and the garland
is ready for the bridegroom.
After the wedding the bride shall leave her home
and meet her lord alone
in the solitude of night. (Paul, 2006, p. 365)

Tagore clearly reflected the idea of facing death with a sense of fulfilment and being prepared for death through these lines. Elsewhere, he described death as a transition from a life filled with activities and responsibilities and desires to the peaceful realm of death, removed from actions and desires:

As the activities of a vigorous vitality may become unmeaning, and thereupon smother the soul with a mere multiplicity of material, so the peace of the extinguished desire may become the peace of death. (Tagore, 1994, p. 486)

These and other references to a sense of fulfilment and peace at the time of death in Indian literature clearly exemplify that being prepared for death was considered as one of the most crucial aspects of the understanding of death. Viewing death as a peaceful end to a complete and fulfilled life, Rabindranath Tagore wrote ‘I know now what death was. It was perfection. Nothing lost’ (Singh, 1983). There are references in the literature to several rituals that were practised in order to help the dying persons achieve peace and fulfilment. For example, in Tamil literature, *Abirami Anthathi*, a compilation of poems by *Abrami Pattar*, has number of poems that give instructions and prayers for chanting next to the dying person in assisting to prepare for death (Pattar, 1977). The prayers in this collection of poems include prayers for the removal of the fear of death, for enabling the detachment from clinging to the body and to the world and to help thinking only about God at the time of death.

Tamil literature from the *Sangam* period, offers another example to demonstrate the importance of fulfilment and detachment in the understanding of death. The period between third-century BCE and fourth-century CE, during which period a lot of poetic and philosophical literature were written. They offer valuable resources regarding the history, social and cultural practices of ancient Tamil Nadu. There are several references in these literature to the practice of ‘Vadakkiruthtal’, where people who have lived a complete life, attained fulfilment and desired death, sat facing the north and fasted until death (Aravanan, 1976). Death in this case was desired by those who have attained fulfilment in their lives and wanted to detach themselves from the embodiment of the human nature and awaited release from the body. They voluntarily deprived themselves of food and water and awaited their own death. The attention to the attainment of the spiritual goals, the sense of fulfilment and detachment from the world and the body, over powers the suffering of the body. Thus, letting go of the body was considered essential and desirable and not a matter of fear (Solayan & Barvin, 2007). Death, therefore, is a process of transition where one moves from this world to be united with the divine with a sense of having attained fulfilment and have detached from this world.

A review of Indian literature on the understanding of death suggests that death is seen as part of human life, just as birth is. However, it does not mark the end of the person. Death marks the end of the embodied nature of the self in this world and the passage to the next form of existence of the ‘self’, the soul. Death of a human person offers the possibility of attaining the ultimate purpose of human life, which is to unite with the Divine. Literature clearly suggested that detachment from the worries and desires of this world and having a sense of fulfilment and peace and God-thought at the time of death are crucial to achieve the ultimate liberation for the soul.

## Conclusion

I have represented significant thoughts on the concept of the human person, the purpose of human life and the meaning of death based on my review of Indian philosophical and Tamil classical literature. The findings reveal that the concept of human person, the understanding of the purpose of human life and the meaning of death were the three conceptual areas that influence the understanding of spirituality at the end of life. Despite differences between the various schools of thoughts all of them present the importance of the soul, the ultimate aim of attaining *moksha* and the requirement of living virtuously in order to be prepared for a death characterised by fulfilment and peace in order to attain the union with the Divine.

Empirical studies on spirituality at the end of life done in India indicate that these Indian philosophical concepts have a crucial influence on the contemporary notions of spirituality at the end of life (Gielen, Bhatnagar, & Chaturvedi, 2017; Simha et al., 2013). Findings of these studies demonstrate the role of religious and philosophical concepts in shaping the participants’ perceptions of spirituality. Conversations in clinical settings often become platforms for patients and their families to express their views and concerns that may contain deep spiritual values. An appreciation of the philosophical foundations and cultural contexts from within which these arise provides the clinicians frameworks to better understand patients’ and families’ experiences and to respond to them appropriately.

Such philosophical explorations are essential to form a comprehensive view of the perceptions of spirituality at the end of life in the Indian setting. Further research is needed to establish how these philosophical foundations continue to shape and influence the spirituality of individuals and communities as they respond to death and dying today. This approach to understanding spirituality provides a contextual frame for understanding the spiritual needs of palliative care patients and their families as well as offers the possibility of developing culturally relevant approaches to providing spiritual care.

## Disclosure statement

No potential conflict of interest was reported by the author.

## Notes on contributor

***Hamilton Inbadas*** is a research associate at the University of Glasgow on the WellcomeTrust funded project, ‘Global Interventions at the End of Life’ [grant number 103319/Z/13/Z] and a member of the Glasgow End of Life Studies Group

## References

[CIT0001] Adikalaar K. (1975). *Religion and society in our view (In Tamil)*. Chennai: Chennai University.

[CIT0002] Adiswarananda S. (2007). *Meditation and its practices: A definitive guide to technniques and traditions of meditation in Yoga and Vedanta*. Woodstock: SkyLight Paths Publishing.

[CIT0003] Aravanan K. (1976). Physical and cultural similarities between Dravidians and Africans. *Journal of Tamil Studies*, 10, 23–27.

[CIT0004] Ārumuganāvalar K. (1987). *Explanation of the unification of the Hindu religion*. Nagercoil: Mārthāndan Kāriyālaya Publications.

[CIT0005] Baba S. S. (1976). *Summer roses on the blue mountains*. Anantapur: Sri Sathya Sai Books and Publications Trust.

[CIT0006] Chakrabarti K. K. (1999). *Classical Indian philosophy of mind: The Nyaya dualist tradition*. New Delhi: Motilal Banarsidass Publishers.

[CIT0007] Chattopadhyay S., & Simon A. (2008). East meets west: Cross-cultural perspective in end-of-life decision making from Indian and German viewpoints. *Medicine, Health Care and Philosophy*, 11, 165–174. 10.1007/s11019-007-9106-y 18046628

[CIT0008] Chattopadhyaya S. K. (2000). *The philosophy of Sankara’s Advaita Vedanta*. New Delhi: Sarup & Sons.

[CIT0009] Cobb M. (2001). *The dying soul: Spiritual care at the end of life*. Buckingham: Open University Press.

[CIT0010] Cobb M., Dowrick C., & Lloyd-Williams M. (2012). What can we learn about the spiritual needs of palliative care patients from the research literature? *Journal of Pain and Symptom Management*, 43, 1105–1119. doi:10.1016/j.jpainsymman.2011.06.017 22575720

[CIT0011] Conze E. (1959). *Buddhist scriptures* (Vol. 88). London: Penguin.

[CIT0012] Dalal N. (2009). Contemplative practice and textual agency in Advaita Vedānta. *Method & Theory in the Study of Religion*, 21, 15–27. 10.1163/157006809X416788

[CIT0013] Dasgupta S. (1922). *The history of Indian philosophy* (Vol. 1). Cambridge: The University Press.

[CIT0014] Davidson G. W. (1988). Hospice care for the dying In Wass H., Berardo F. M., & Neimeyer R. A. (Ed.), *Dying: Facing the facts* (2 ed., pp. 185–200). Washington: Hemisphere Publishing Corporation.

[CIT0015] Desai P. N. (1988). Medical ethics in India. *Journal of Medicine and Philosophy*, 13, 231–255. 10.1093/jmp/13.3.231 3058850

[CIT0016] Elsner F., Schmidt J., Rajagopal M., Radbruch L., & Pestinger M. (2012). Psychosocial and spiritual problems of terminally ill patients in Kerala, India. *Future Oncology*, 8, 1183–1191. 10.2217/fon.12.97 23030492

[CIT0017] Eseadi C., Onwuasoanya P. N., Ikechukwu-Ilomuanya A. B., Ogbuabor S. E., Otu M. S., & Ede M. O. (2015). Religiotherapy: A Panacea for incorporating religion and spirituality in counselling relationship. *Journal of Research & Method in Education*, 5, 64–77.

[CIT0018] Fegg M. J., Brandstätter M., Kramer M., Kögler M., Haarmann-Doetkotte S., & Borasio G. D. (2010). Meaning in life in palliative care patients. *Journal of Pain and Symptom Management*, 40, 502–509. 10.1016/j.jpainsymman.2010.02.010 20594803

[CIT0019] Firth S. (2005). End-of-life: A Hindu view. *The Lancet*, 366, 682–686. 10.1016/S0140-6736(05)67141-3 16112306

[CIT0020] Ganapathy T. N. (1993). *The philosophy of the Tamil Siddhas*. Chennai: Ravi publications.

[CIT0021] Ganeri J. (2007). *The concealed art of the soul: Theories of self and practices of truth in Indian ethics and epistemology*. Oxford: Oxford University Press 10.1093/acprof:oso/9780199202416.001.0001

[CIT0022] Ghosh S. (2014). Duty, consequences, and intellectual property. *University of St. Thomas Law Journal*, 10, 10.

[CIT0023] Gielen J., Bhatnagar S., & Chaturvedi S. K. (2016). Spirituality as an ethical challenge in Indian palliative care: A systematic review. *Palliative and Supportive Care*, 14, 561–582. doi:10.1017/s147895151500125x 26510891

[CIT0024] Gielen J., Bhatnagar S., & Chaturvedi S. K. (2017). Prevalence and nature of spiritual distress among palliative care patients in India. *Journal of Religion and Health*, 56, 530–544. doi:10.1007/s10943-016-0252-5 27154352

[CIT0025] Goddard N. C. (1995). ‘Spirituality as integrative energy’: A philosophical analysis as requisite precursor to holistic nursing practice. *Journal of Advanced Nursing*, 22, 808–815. 10.1046/j.1365-2648.1995.22040808.x 8708203

[CIT0026] Gupta B. (2002). *Ethical questions: East and west*. Maryland: Rowman & Littlefield.

[CIT0027] Henery N. (2003). Constructions of spirituality in contemporary nursing theory. *Journal of Advanced Nursing*, 42, 550–557. 10.1046/j.1365-2648.2003.02658.x 12787228

[CIT0028] Hermsen M. A., & ten Have H. A. (2004). Pastoral care, spirituality, and religion in palliative care journals. *American Journal of Hospice and Palliative Medicine*, 21, 353–356. 10.1177/104990910402100509 15510572

[CIT0029] Hiriyanna M. (1995). *The essentials of Indian philosophy*. New Delhi: Motilal Banarsidass Publ.

[CIT0030] Inbadas H. (2016). History, culture and traditions: The silent spaces in the study of spirituality at the end of life. *Religions*, 7, 53 10.3390/rel7050053

[CIT0031] Iyengar B. K. S. (2004). *Light on the yoga sutras of Patanjali*. Columbia: South Asia Books.

[CIT0032] Kellehear A. (2000). Spirituality and palliative care: A model of needs. *Palliative Medicine*, 14, 149–155. 10.1191/026921600674786394 10829149

[CIT0033] Kesavan H. (1997). *The Vedas and Vedic philosophy*. In Sridhar S. N. and Mattoo Nirmal K. (Eds.), *Ananya: A portrait of India* (pp. 137–156). Rockville: Association of Indians in America.

[CIT0034] King R. (1999). *Indian philosophy: An introduction to Hindu and Buddhist thought*. Edinburgh: Edinburgh University Press.

[CIT0035] Konwar B. (2013). Vivekananda’s concept of karma. *Social Science Researcher*, 2, 16–21.

[CIT0036] Krishnananda S. (1977). *The Chhandogya upanishad*. Rishikesh: The Divine Life Society.

[CIT0037] Kutumbiah P. (1999). *Ancient Indian medicine*. Hyderabad: Orient Blackswan.

[CIT0038] Lal B. K. (1978). *Contemporary Indian philosophy*. Delhi: Motilal Banarsidass.

[CIT0039] Mahadevan T. M. P. (1953). The religio-philosophic culture of India In Bhattacharyya H. & Mission R. (Eds.), *The cultural heritage of India* (2 ed., Vol. 1, pp. 163–181). Calcutta: Ramakrishna Mission, Institute of Culture.

[CIT0040] Mahalingam I. (2002). Sā Khya-Yoga In Carr B. & Mahalingam I. (Ed.), *Companion encyclopaedia of Asian philosophy* (pp. 139–154). London: Routledge.

[CIT0041] Manghrani N., & Kapadia S. (2006). *Death and dying: Strategies for improving quality of life of terminally ill patients in India*. Paper presented at the Strength Based Strategies, Hydrabad.

[CIT0042] Mehta J. (2013). Palliative health care ancient wisdom. *American Journal of Hospice and Palliative Medicine*, 30, 512–513. 10.1177/1049909112453081 22811211

[CIT0043] Mohanty J. N. (1993). *Essays on Indian philosophy traditional and modern*. Bombay: Oxford University Press.

[CIT0044] Mohanty J. N. (2001). *Indian philosophy*. New Delhi: Oxford University Press.

[CIT0045] Monier-Williams M. (1974). *Religious thought and life in India: Vedism, Brahmanism and Hinduism*. London: Oriental Books Reprint.

[CIT0046] Narayanasamy A. (2006). *Spiritual care and transcultural care research*. Dinton: Quay Books.

[CIT0047] Nilakanta Sāstri K. A. (1972). *Sangam literature: Its cults and cultures*. Madras: Swathi Publications.

[CIT0048] Pattar A. (1977). *Abirami Anthathi (in Tamil)*. Chennai: Amuthanilayam Limited.

[CIT0049] Patterson E. F. (1998). The philosophy and physics of holistic health care: Spiritual healing as a workable interpretation. *Journal of Advanced Nursing*, 27, 287–293. 10.1046/j.1365-2648.1998.00533.x 9515638

[CIT0050] Paul S. K. (2006). *The complete poems of Rabindranath Tagore’s Gitanjali: Texts and critical evaluation*. New Delhi: Sarup & Sons.

[CIT0051] Pesut B., & Sawatzky R. (2006). To describe or prescribe: Assumptions underlying a prescriptive nursing process approach to spiritual care. *Nursing Inquiry*, 13, 127–134. doi:10.1111/j.1440-1800.2006.00315.x 16700756

[CIT0052] Prabhupada A. C. B. S. (1983). *Bhagavad-Gita as it is*. Los Angeles: The Bhaktivedanta Book Trust.

[CIT0053] Prasad R. (2008). *A conceptual-analytic study of classical Indian philosophy of morals*. New Delhi: Concept Publishing Company.

[CIT0054] Reddiyar S. (2001). *Sura’s Thirukkural and explanation*. Chennai: Sura Books.

[CIT0055] Śankarāchārya Ś. (1983). *Bhagavad Gītā Bhāșya*. Madras: Sri Ramakrishna Math.

[CIT0056] Simha S., Noble S., & Chaturvedi S. K. (2013). Spiritual concerns in Hindu cancer patients undergoing palliative care: A qualitative study. *Indian Journal of Palliative Care*, 19, 99–105. 10.4103/0973-1075.116716 24049350 PMC3775032

[CIT0057] Singh J. (1983). *Dictionary of Indian quotations*. New Delhi: Parichay Overseas.

[CIT0058] Solayan M., & Barvin A. M. (2007). The concept of death in Cankam and English metaphysical poetry. *Journal of Tamil Studies* (71), 88–92.

[CIT0059] Subramaniyan K. (1982). *Society in the Sangam period (in Tamil)*. Chennai: New Century Publications.

[CIT0060] Swinton J. (2010). The meaning of spirituality: A multi-perspective approach to ‘the spiritual’ In McSherry W. & Ross L. A. (Eds.), *Spiritual assessment in healthcare practice* (pp. 17–36). Keswick: M&K Update Ltd.

[CIT0061] Swinton J., & Narayanasamy A. (2002). Response to: ‘A critical view of spirituality and spiritual assessment’ by P. Draper and W. McSherry (2002) Journal of Advanced Nursing 39, 1–2. *Journal of Advanced Nursing*, 40, 158–160. 10.1046/j.1365-2648.2002.02401.x 12366645

[CIT0062] Tagore R. (1994). *The English writings of Rabindranath Tagore: A miscellany* (Vol. 3). New Delhi: Sahitya Akademi.

[CIT0063] Vade V. B. (2002). *Value perspectives in Indian philosophy*. New Delhi: Mittal Publications.

[CIT0064] Vivekananda S. (1991). *The complete work of Swami Vivekananda* (Vol. 2). Calcutta: Advaita Ashrama.

